# Development of diagnostic algorithm using machine learning for distinguishing between active tuberculosis and latent tuberculosis infection

**DOI:** 10.1186/s12879-022-07954-7

**Published:** 2022-12-29

**Authors:** Ying Luo, Ying Xue, Wei Liu, Huijuan Song, Yi Huang, Guoxing Tang, Feng Wang, Qi Wang, Yimin Cai, Ziyong Sun

**Affiliations:** 1 Department of Laboratory Medicine, Tongji Hospital, Tongji Medical College, Huazhong University of Science and Technology, Jiefang Road 1095, Wuhan, 430030 China; 2 Department of Immunology, School of Basic Medicine, Tongji Medical College, Huazhong University of Science and Technology, Hangkong Road 13, Wuhan, China; 3 Télécom Physique Strasbourg, Illkirch-Graffenstaden, France; 4 Department of Epidemiology and Biostatistics, Key Laboratory of Environmental Health of Ministry of Education, School of Public Health, Tongji Medical College, Huazhong University of Science and Technology, Hangkong Road 13, Wuhan, China

**Keywords:** Diagnostic algorithm, Machine learning, Active tuberculosis, Latent tuberculosis infection, Discrimination

## Abstract

**Background:**

The discrimination between active tuberculosis (ATB) and latent tuberculosis infection (LTBI) remains challenging. The present study aims to investigate the value of diagnostic models established by machine learning based on multiple laboratory data for distinguishing *Mycobacterium tuberculosis* (Mtb) infection status.

**Methods:**

T-SPOT, lymphocyte characteristic detection, and routine laboratory tests were performed on participants. Diagnostic models were built according to various algorithms.

**Results:**

A total of 892 participants (468 ATB and 424 LTBI) and another 263 participants (125 ATB and 138 LTBI), were respectively enrolled at Tongji Hospital (discovery cohort) and Sino-French New City Hospital (validation cohort). Receiver operating characteristic (ROC) curve analysis showed that the value of individual indicator for differentiating ATB from LTBI was limited (area under the ROC curve (AUC) < 0.8). A total of 28 models were successfully established using machine learning. Among them, the AUCs of 25 models were more than 0.9 in test set. It was found that conditional random forests (cforest) model, based on the implementation of the random forest and bagging ensemble algorithms utilizing conditional inference trees as base learners, presented best discriminative power in segregating ATB from LTBI. Specially, cforest model presented an AUC of 0.978, with the sensitivity of 93.39% and the specificity of 91.18%. Mtb-specific response represented by early secreted antigenic target 6 (ESAT-6) and culture filtrate protein 10 (CFP-10) spot-forming cell (SFC) in T-SPOT assay, as well as global adaptive immunity assessed by CD4 cell IFN-γ secretion, CD8 cell IFN-γ secretion, and CD4 cell number, were found to contribute greatly to the cforest model. Superior performance obtained in the discovery cohort was further confirmed in the validation cohort. The sensitivity and specificity of cforest model in validation set were 92.80% and 89.86%, respectively.

**Conclusions:**

Cforest model developed upon machine learning could serve as a valuable and prospective tool for identifying Mtb infection status. The present study provided a novel and viable idea for realizing the clinical diagnostic application of the combination of machine learning and laboratory findings.

**Supplementary Information:**

The online version contains supplementary material available at 10.1186/s12879-022-07954-7.

## Highlights

The first study to establish 28 models using machine learning for TB diagnosis.

The first TB diagnostic model based on routine, TB-specific and non-specific tests.

Cforest model presented excellent performance in discriminating ATB from LTBI.

**Supplementary Information:**

The online version contains supplementary material available at 10.1186/s12879-022-07954-7.

## Introduction

Tuberculosis (TB), caused by *Mycobacterium tuberculosis* (Mtb) infection, is one of the leading contagious diseases globally, with approximately 10.6 million new cases and 1.6 million deaths in 2021 [1]. Individuals infected with Mtb can be classified into active TB (ATB) and latent TB infection (LTBI) based on their clinical manifestations [2]. The accurate and rapid differential diagnosis between these two states is essential for TB management and the final realization of ending TB [3–5]. Currently, identifying Mtb infection status remains an issue despite intensive achieved efforts [6, 7]. Therefore, the development of novel and effective diagnostic strategies should be a strategic priority in combating the disease.

Existing gold-standard approaches, including acid-fast staining, mycobacterial culture, and molecular tests, failed to meet clinical needs for TB diagnostics due to either limited sensitivity or time-consuming [8]. Many emerging omics-based approaches including transcriptomics [9, 10], proteomics [11, 12], and metabolomics [13, 14], have been developed for TB diagnostics. Nevertheless, these tests are currently unable to be applied into clinical practice as a consequence of high dependence of instrument, poor reproducibility, and the lack of wide-range validation [15].

The delay in TB diagnosis was probably partially bridled by insufficient use of obtained data from laboratory. Studies from many teams and our own pervious investigation demonstrated that the diagnostic value of data from routine laboratory tests should not be neglected. Laboratory data revealing host characteristics in different dimensions have potential for the diagnosis of TB [16, 17]. Results from blood examination, biochemical tests, coagulation detection, and T-SPOT assay showed mediocre value in identifying Mtb infection status [18, 19]. In addition, the value of the detection targeting lymphocyte number and function for TB diagnostics was also confirmed by two recent reports [20, 21]. Although these tests were of limited discriminatory value when they were used separately, the diagnostic performance of these data could be effectively improved when the data is integrated with appropriate algorithm. The rapid development of artificial intelligence has given a lot of emerging opportunities to laboratory data for this purpose. In this study, we developed diagnostic algorithm using machine learning based on multiple-test data for distinguishing ATB from LTBI and validated it.

## Methods

### Study design

The current study was carried out from January 2018 to January 2022. The study participants in discovery cohort were recruited at Tongji Hospital (the largest tertiary hospital in central China with 5500 beds). The study participants in validation cohort were enrolled at Sino-French New City Hospital (a branch hospital of Tongji Hospital with 1600 beds). Participants in two cohorts were included based on positive T-SPOT results. Participants were classified as ATB patients and LTBI individuals on the grounds of clinical and laboratory evaluation. ATB was diagnosed by positive Mtb culture and/or GeneXpert MTB/RIF for the allocated samples including bronchoalveolar lavage fluid and sputum. LTBI was defined by positive T-SPOT result without symptomatic, radiological or microbiological evidences of ATB as well as the history of TB. Specially, the symptoms compatible of ATB in the current study included prolonged cough, chest pain, fever, and night sweats. Patients with the following condition were excluded from the study: (1) having anti-TB treatment within 1 month prior to the enrollment; (2) being younger than 18 years old. This study was approved by the ethics committee of Tongji Hospital, Tongji Medical College, Huazhong University of Science and Technology.

### Routine laboratory tests

Blood routine examination and detection of biochemical, coagulation and inflammatory indicators were performed on each participant. Briefly, ethylenediaminetetraacetic acid-anticoagulated peripheral blood was collected for routine blood examination. The following indicators were obtained: white blood cell count (WBC), neutrophil count (NEUT), lymphocyte count (LYMPH), monocyte count (MONO), eosinophil count (EO), basophil count (BASO), red blood cell count (RBC), hemoglobin (HGB), hematocrit (HCT), coefficient variation of red blood cell volume distribution width (RDW_CV), standard deviation in red cell distribution width (RDW_SD), platelet count (PLT), platelet larger cell ratio (P_LCR), plateletcrit (PCT), and platelet distribution width (PDW). Heparin anticoagulating peripheral blood was collected for biochemical indicators detection. The following parameters were obtained: total protein (TP), albumin (ALB), globulin (GLB), total cholesterol (T_CHOL), triglyceride (TG), calcium (Ca), chlorine (Cl), kalium (K), natrium (Na), phosphor (P), magnesium (Mg), and hypersensitive C-reactive protein (HsCRP). Sodium citrate anti-coagulated peripheral blood was collected for coagulation indicator detection. The following indexes were obtained: activated partial thromboplastic time (APTT), fibrinogen (FIB), prothrombin time (PT), thrombin time (TT), d-dimer (D_D), and erythrocyte sedimentation rate (ESR). The used instruments included XN-9000 Sysmex (Sysmex Co., Kobe, Japan), ROCHE COBAS (Mannheim, Germany), STA-R coagulation analyzers (Diagnostic Stago, France), and Monitor100 (SYSMEX).

### T-SPOT

Heparin anticoagulated peripheral blood was collected for T-SPOT assay (Oxford Immunotec, Oxford, UK). Briefly, the isolated peripheral blood mononuclear cells (PBMCs) (2.5 × 10^5^) were added to 96-well plates precoated with anti-IFN-γ antibody. Four wells were prepared for the test: medium, early secreted antigenic target 6 (ESAT-6), and culture filtrate protein 10 (CFP-10), phytohemagglutinin (PHA). Plates were incubated for 16–20 h at 37 °C with 5% CO_2_ and developed using an anti-IFN-γ antibody conjugate and substrate to detect the presence of secreted IFN-γ. Spot-forming cell (SFC) in each well was counted by ELISPOT reader (CTL Analyzers, Cleveland, OH, USA). The result was regarded as positive when ESAT-6 minus medium or CFP-10 minus medium ≥ 6. The result was regarded as negative if both ESAT-6 minus medium and CFP-10 minus medium ≤ 5. The result was considered as undetermined when the spot number in PHA well was < 20 or spot number in medium well was > 10.

### Lymphocyte subset number and IFN-γ secretion ability detection

Heparinized peripheral blood was collected for the measurement of lymphocyte subset number and lymphocyte IFN-γ secretion ability. The numbers of CD4^+^ T cells, CD8^+^ T cells, NK cells, and B cells were determined by using TruCOUNT tubes and BD lymphocyte subset reagent kit (BD Biosciences, San Jose, CA, USA). A volume of 50 µL peripheral blood was labeled with antibody cocktail for 20 min in room temperature. After adding 450 µL of FACS lysing solution, samples were analyzed with FACSCanto flow cytometer. TruCOUNT beads were gated based on side scatter and fluorescence intensity. CD3^+^CD4^+^CD8^−^ and CD3^+^CD4^−^CD8^+^ cells were respectively defined as CD4^+^ T cells and CD8^+^ T cells. CD16^+^CD56^+^ cells and CD19^+^ cells in CD3^−^ cells were respectively defined as NK cells and B cells. Lymphocyte IFN-γ secretion ability detection was performed under phorbol-12-myristate-13-acetate/Ionomycin/ionomycin (PMA/Ionomycin) stimulation as described in previous study [22]. The procedure was as the following: (1) 100 µL peripheral blood was diluted with 400 µL of IMDM medium (Gibco, Grand Island, NY, USA); (2) the diluted peripheral blood was incubated in the presence of Leukocyte Activation Cocktail (Becton Dickinson GolgiPlug™) for 4 h; (3) the cells were labeled with antibodies including anti-CD45, anti-CD3, anti-CD4, anti-CD8, and anti-CD56 for 20 min at room temperature; (4) the cells were fixed and permeabilized; (5) the cells were stained with intracellular anti-IFN-γ antibody; and (6) the cells were analyzed with FACSCanto flow cytometer. The percentages of IFN-γ^+^ cells in cell subsets were defined as IFN-γ secretion ability of them. Specially, the percentage of IFN-γ^+^ cells in CD3^+^CD4^+^CD8^−^ cells was regarded as CD4^+^ T cell IFN-γ secretion ability; the percentage of IFN-γ^+^ cells in CD3^+^CD4^−^CD8^+^ cells was regarded as CD8^+^ T cell IFN-γ secretion ability; the percentage of IFN-γ^+^ cells in CD3^−^CD56^+^ cells was regarded as NK cell IFN-γ secretion ability.

### Establishment of diagnostic models

Diagnostic models were established using machine learning by the R package “mlr3” and related packages. Multiple data acquired from study participants in discovery cohort was randomly divided at a 3:1 ratio. The large one (3/4) was utilized for modelling (training set), whereas the small one (1/4) was applied as test set. The models established in discovery cohort were further verified using an independent cohort (validation set). Machine learning learners used were generated using R packages “mlr3”, “mlr3learners”, and “mlr3extralearners”. The probability ranging between 0 and 1 for ATB diagnosis for each case was obtained by the prediction of the model. The performance of models was evaluated by measures involved in R package “mlr3”. The importance of indicators in the contribution to the model was also evaluated.

### Statistical analysis

Continuous variables were represented as mean ± standard deviation (SD) or medians. Categorical variables were expressed as number (%). Student’s *t* test and Mann–Whitney *U* test were applied for the comparison of continuous variables. Chi-square test and Fisher’s exact test were used for the comparison of categorical variables. *P* < 0.05 represented that statistical difference existed. Cor linear regression was performed to evaluate whether there is a linear correlation between various indicators. Tree-leaf clustering, principal components analysis (PCA), t-distributed stochastic neighbor embedding (t-SNE), and uniform manifold approximation and projection (UMAP) were utilized to visualize the differentiation of multiple results. Receiver operating characteristic (ROC) curves were created to evaluate the performance of various indicators and models for discriminating ATB from LTBI. Area under the ROC curve (AUC), sensitivity, specificity, positive predictive value (PPV), negative predictive value (NPV), positive likelihood ratio (PLR), negative likelihood ratio (NLR), as well as accuracy, together with their 95% confidence intervals (CI), were calculated. The comparison between AUCs was achieved by DeLong’s test [23]. The tools involved in data analysis and graphing throughout the study included R 4.0.2 program (R Core Team), GraphPad Prism Software 6.0 (GraphPad Software, Inc, San Diego, CA, USA), Java (TM) SE Development Kit 11.0.14 (Oracle), SPSS Software 25.0 (Social Sciences Inc, Chicago, Illinois, USA), and MedCalc 11.6 (MedCalc, Mariakerke, Belgium).

## Results

### Characteristics of recruited participants

A total of 468 patients with ATB and 424 individuals with LTBI were recruited in discovery cohort, while 125 patients with ATB and 138 individuals with LTBI were enrolled in validation cohort (Table 1). There is a preponderance of male cases in both ATB group and LTBI group. Diabetes mellitus is the major underlying disease in both two groups. There was no significant difference in the age and sex distribution between ATB group and LTBI group in both discovery and validation cohorts.

**Table 1:** Demographic and clinical characteristics of the recruited participants

Variables	Discovery cohort		P*	Validation cohort		P*	*P*^†^
	ATB (n = 468)	LTBI (n = 424)		ATB (n = 125)	LTBI (n = 138)		
Age, years	52.38 ± 14.04	53.08 ± 14.47	0.573	51.70 ± 13.68	53.51 ± 13.59	0.209	0.785
Sex, male, %	289 (61.75%)	247 (58.25%)	0.287	81 (64.80%)	83 (60.14%)	0.436	0.508
Underlying condition or illness							
Diabetes mellitus	92 (19.66%)	77 (18.16%)	0.569	27 (21.60%)	24 (17.39%)	0.389	0.872
Virus hepatitis or cirrhosis	52 (11.11%)	39 (9.20%)	0.346	16 (12.80%)	16 (11.59%)	0.765	0.364
Nephritis or renal failure	36 (7.69%)	22 (5.19%)	0.13	8 (6.40%)	11 (7.97%)	0.623	0.68
Solid tumor	30 (6.41%)	27 (6.37%)	0.979	7 (5.60%)	9 (6.52%)	0.755	0.858
Heart disease	29 (6.20%)	18 (4.25%)	0.193	6 (4.80%)	6 (4.35%)	0.861	0.648
Positive culture for Mtb	398 (85.04%)	N/A	N/A	112 (89.60%)	N/A	N/A	N/A
Positive GeneXpert MTB/RIF	381 (81.41%)	N/A	N/A	106 (84.80%)	N/A	N/A	N/A

ATB: active tuberculosis; LTBI: latent tuberculosis infection; Mtb: *Mycobacterium tuberculosis*; N/A: not applicable. *Comparisons were performed between ATB and LTBI groups using Mann–Whitney *U* test or Chi-square test. ^†^Comparisons were performed between discovery cohort and validation cohort using Mann–Whitney *U* test or Chi-square test. Data were presented as means ± standard deviation or numbers (percentages)

### Performance of individual indicators for distinguishing ATB from LTBI

**Fig. 1:**
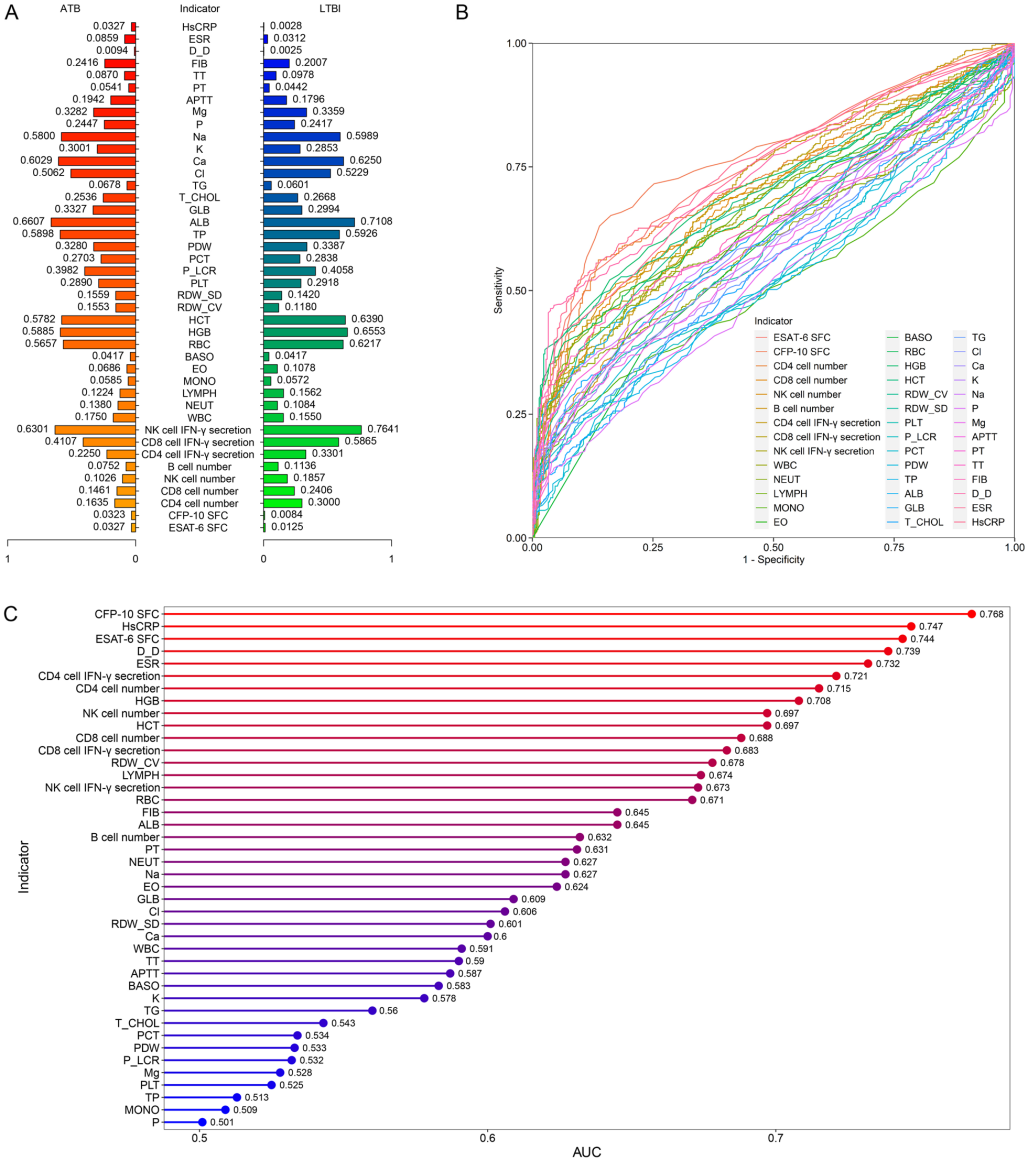
The performance of various indicators in distinguishing between ATB patients and LTBI individuals. **A** Pyramid delineating the comparison of various indicators between ATB patients and LTBI individuals. The values represented the median after normalization to range between 0 and 1. **B** ROC curves showing the performance of individual indicators in segregating ATB patients from LTBI individuals. **C** Cleveland dot plot showing the AUCs of various indicators in discriminating ATB patients from LTBI individuals. ATB: active tuberculosis; LTBI: latent tuberculosis infection; ROC: receiver operator characteristics; AUC: area under the ROC curve

Most indicators showed significant differences between ATB patients and LTBI individuals. It was observed that the levels of ESAT-6 SFC, CFP-10 SFC, WBC, NEUT, RDW_CV, RDW_SD, GLB, TG, K, APTT, PT, FIB, D_D, ESR, and HsCRP were significantly higher in ATB patients than those in LTBI individuals (Fig. 1A). On the contrary, the levels of CD4 cell number, CD8 cell number, NK cell number, B cell number, CD4 cell IFN-γ secretion, CD8 cell IFN-γ secretion, NK cell IFN-γ secretion, LYMPH, EO, BASO, RBC, HGB, HCT, ALB, T_CHOL, Cl, Ca, Na, and TT were significantly lower in ATB patients than those in LTBI individuals (Fig. 1A). There was no statistical difference in the levels of MONO, PLT, P_LCR, PCT, PDW, TP, P, and Mg between ATB patients and LTBI individuals. The capability of individual indicator to distinguish ATB patients from LTBI individuals was determined using ROC curve analysis. It was found that the AUCs of 8 indicators were more than 0.7, while the AUCs of the remaining 34 indicators were under 0.7 (Fig. 1B, C). Specially, CFP-10 SFC, HsCRP, ESAT-6 SFC, D_D, ESR, CD4 cell IFN-γ secretion, CD4 cell number, and HGB were the most accurate biomarkers in differentiating ATB from LTBI (Fig. 1B, C).

### Establishing diagnostic models using machine learning

**Fig. 2:**
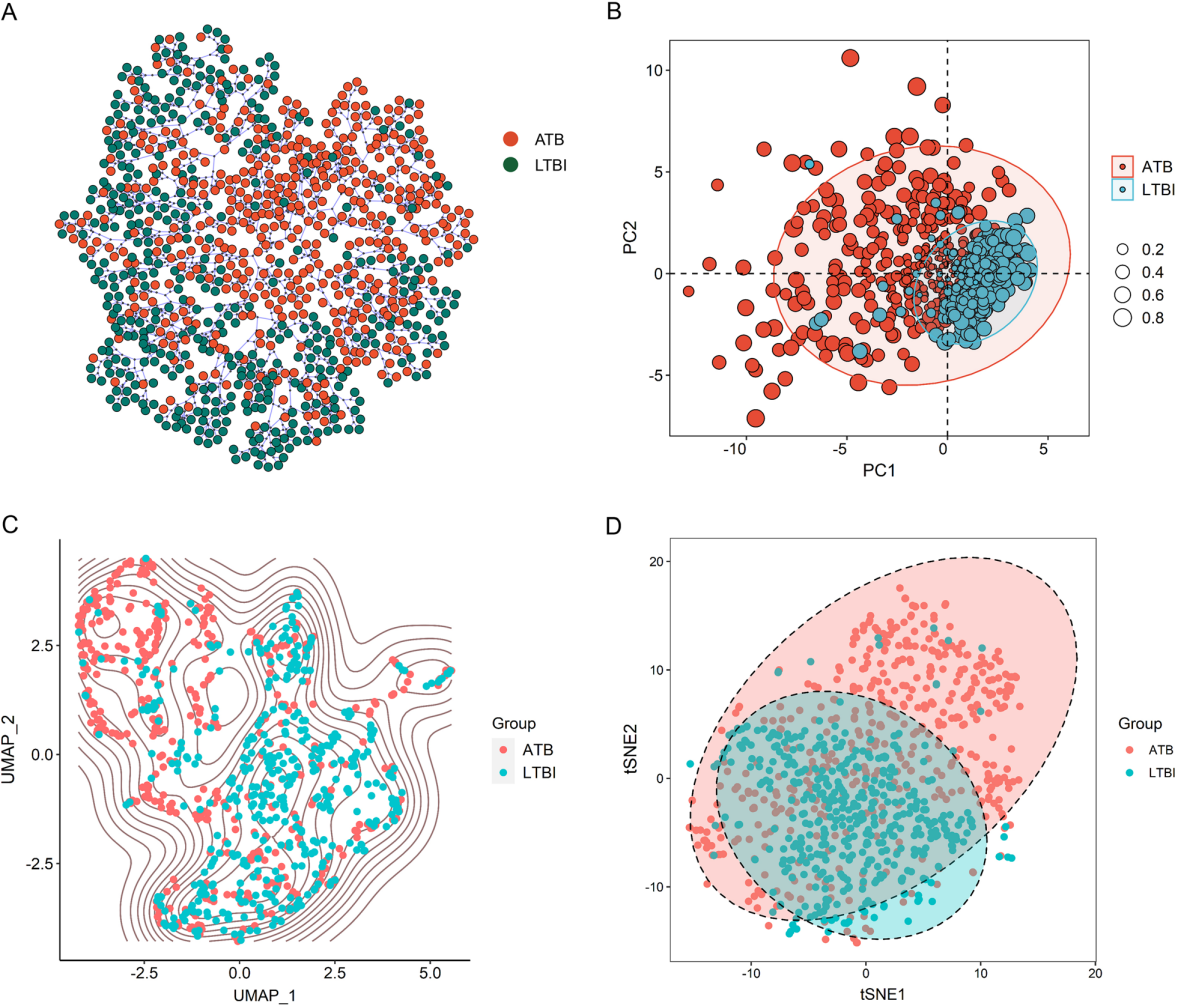
Clustering and dimension reduction analysis based on laboratory data of ATB patients and LTBI individuals. **A** Tree and leaf plots showing the clustering on the basis of laboratory data. **B** The plot showing PCA dimension reduction based on laboratory data. The size of the circle represents the cos2. **C** The plot showing UMAP dimension reduction based on laboratory data. **D** The plot showing tSNE dimension reduction based on laboratory data. ATB: active tuberculosis; LTBI: latent tuberculosis infection; PCA: principal components analysis; tSNE: t-distributed stochastic neighbor embedding; UMAP: uniform manifold approximation and projection

Given the fact that the combination of various biomarkers has shown better performance than single biomarker in TB diagnostic field, we attempted to investigate the diagnostic potential of the combination of multiple indicators using machine learning. Cluster analysis and dimension reduction were applied to evaluate the distribution of ATB patients and LTBI individuals based on various indicators. leaf clustering advocated the possibility of the combination of these indicators for the discrimination between ATB and LTBI (Fig. 2A). We further conducted dimension reduction. Consistent with leaf clustering, dimension reduction performed by PCA, tSNE and UMAP analysis also corroborated that the multiple data had the potential to segregate ATB from LTBI (Fig. 2B–D).

**Fig. 3:**
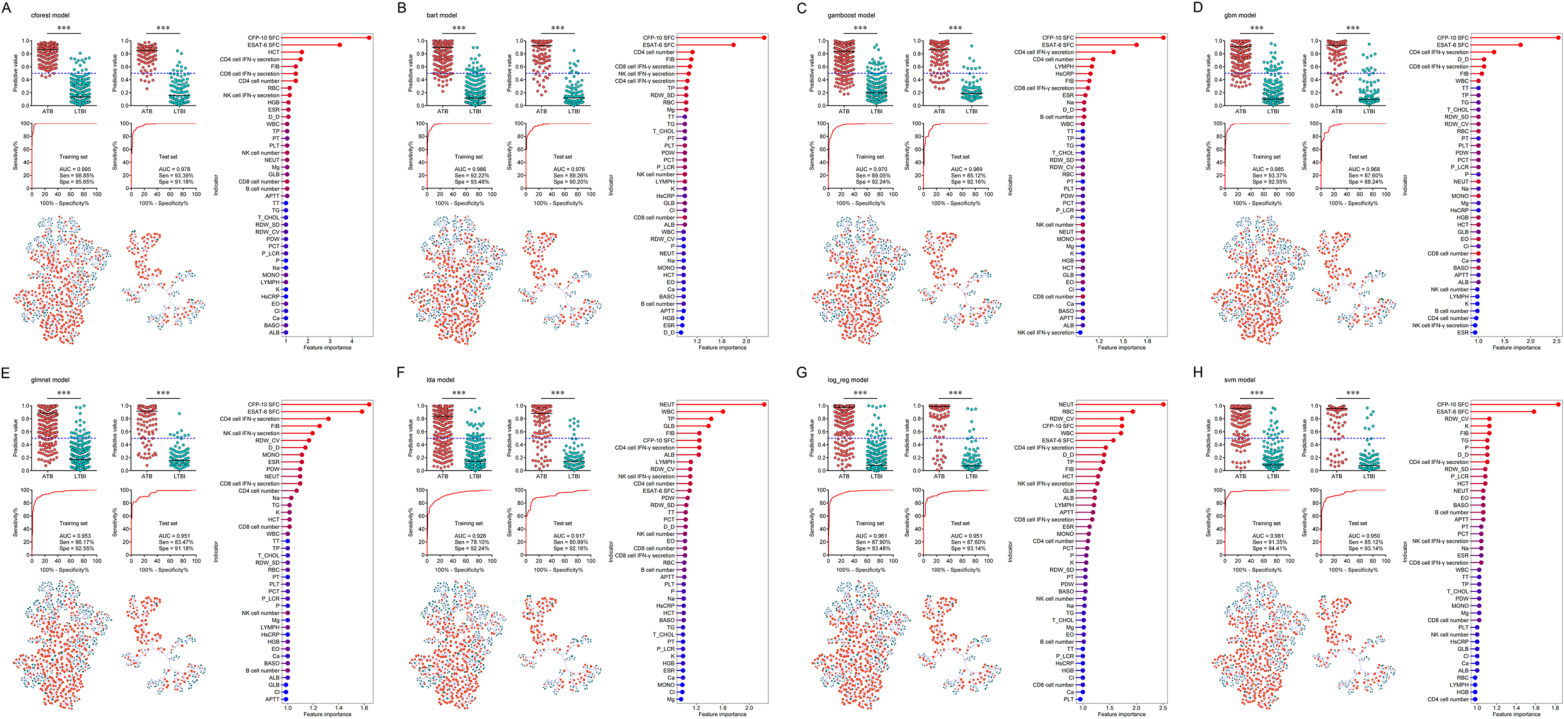
The performance of different diagnostic models established by machine learning for discriminating ATB patients from LTBI individuals in discovery cohort. Scatter plots showing predictive values of diagnostic models (**A** cforest; **B** bart; **C** gamboost; **D** gbm; **E** glmnet; **F** lda; **G** log_reg; **H** svm) in ATB patients and LTBI individuals. Horizontal lines indicate the median. ***P < 0.001 (Mann–Whitney *U* test). Blue dotted lines indicate the cutoff value (0.5) in segregating these two groups. ROC curves showing the performance of diagnostic models (**A** cforest; **B** bart; **C** gamboost; **D** gbm; **E** glmnet; **F** lda; **G** log_reg; **H** svm) in segregating ATB patients from LTBI individuals. Tree and leaf plots showing predictive value of each participant when displaying as cluster distribution. The size of circle represents the predictive value. Cleveland dot plot showing the importance of various indicators in contributing to the model. ATB: active tuberculosis; LTBI: latent tuberculosis infection; ROC: receiver operator characteristics; AUC: area under the ROC curve

Based on the synergistic effects of various indicators denoted by the above findings, 28 diagnostic models were successfully established using machine learning in accordance with laboratory data. ROC curve analysis was performed and the results demonstrated that most established models could successfully differentiate ATB from LTBI with AUCs more than 0.9. Among them, conditional random forests (cforest) model performed better in comparison to other models. Cforest model is an implementation of the random forest and bagging ensemble algorithms utilizing conditional inference trees as base learners. The cforest algorithm could use multiple decision trees to achieve a robust prediction. The model could also avoid overfitting issue since it takes the average and cancels out the biases. ROC curve analysis provided an AUC of 0.995 (95% CI 0.991–0.998) for cforest model in training set (n = 669, 347 ATB and 322 LTBI). The cutoff of 0.5 rendered 98.85% (95% CI 97.07–99.55%) sensitivity and 95.65% (95% CI 92.84–97.39%) specificity. Meanwhile, cforest model differentiated ATB from LTBI with an AUC of 0.978 (95% CI 0.962–0.993) corresponding to a sensitivity of 93.39% (95% CI 87.50–96.61%) and specificity of 91.18% (95% CI 84.08–95.29%) in test set (n = 223, 121 ATB and 102 LTBI) (Table 2, Fig. 3A). CFP-10 SFC, ESAT-6 SFC, HCT, CD4 cell IFN-γ secretion, FIB, CD8 cell IFN-γ secretion, and CD4 cell number were the indicators with the highest contribution to cforest model (Fig. 3A). Among these parameters, CFP-10 SFC and ESAT-6 SFC indicated the specific response of the host against Mtb. In addition, CD4 cell IFN-γ secretion, CD8 cell IFN-γ secretion, and CD4 cell number indicated the global adaptive immunity of the host. Apart from cforest model, other models also showed effective discriminatory value. For example, the sensitivity and specificity of bart model in test set were 89.26% (95% CI 82.48–93.61%) and 90.20% (95% CI 82.89–94.59%), respectively (Table 2, Fig. 3B). Gamboost model distinguished patients with ATB from those with LTBI with an AUC of 0.969 (95% CI 0.949–0.988) and demonstrated a sensitivity of 85.12% (95% CI 77.71–90.38%) and specificity of 92.16% (95% CI 85.28–95.97%) in test set (Table 2, Fig. 3C). Besides, the sensitivity and specificity for gbm model in test set were 87.60% (95% CI 80.55–92.34%) and 88.24% (95% CI 80.55–93.14%), with an AUC of 0.968 (95% CI 0.949–0.987) (Table 2, Fig. 3D). Meanwhile, log_reg model established based on logistic regression had an AUC of 0.951 (95% CI 0.924–0.978) for discriminating ATB patients from LTBI individuals in test set (Table 2, Fig. 3G). The sensitivity and specificity of log_reg model were 87.60% (95% CI 80.55–92.34%) and 93.14% (95% CI 86.51–96.64%), respectively (Table 2, Fig. 3G). The AUCs of ROC curves of various models for ATB versus LTBI were presented in Fig. 3. The performance parameters for all models in training set and test set were shown in Fig. 5A, B and Additional file 1: Fig. S1A-B.

**Table 2:** The performance of various models for segregating ATB from LTBI in discovery cohort

Parameters	Training set (n = 669, 347 ATB, 322 LTBI)								Test set (n = 223, 121 ATB, 102 LTBI)							
	cforest	bart	gamboost	gbm	glmnet	lda	log_reg	svm	cforest	bart	gamboost	gbm	glmnet	lda	log_reg	svm
AUC (95% CI)	0.995 (0.991–0.998)	0.986 (0.979–0.992)	0.970 (0.959–0.981)	0.985 (0.978–0.992)	0.953 (0.937–0.968)	0.926 (0.907–0.946)	0.961 (0.947–0.975)	0.981 (0.972–0.990)	0.978 (0.962–0.993)	0.976 (0.961–0.991)	0.969 (0.949–0.988)	0.968 (0.949–0.987)	0.951 (0.926–0.977)	0.917 (0.880–0.954)	0.951 (0.924–0.978)	0.950 (0.924–0.976)
Sensitivity (95% CI)	98.85% (97.07–99.55%)	92.22% (88.92–94.60%)	89.05% (85.33–91.92%)	93.37% (90.25–95.54%)	86.17% (82.14–89.41%)	78.10% (73.45–82.13%)	87.90% (84.04–90.92%)	91.35% (87.93–93.88%)	93.39% (87.50–96.61%)	89.26% (82.48–93.61%)	85.12% (77.71–90.38%)	87.60% (80.55–92.34%)	83.47% (75.84–89.04%)	80.99% (73.09–86.99%)	87.60% (80.55–92.34%)	85.12% (77.71–90.38%)
Specificity (95% CI)	95.65% (92.84–97.39%)	93.48% (90.24–95.70%)	92.24% (88.79–94.69%)	92.55% (89.15–94.94%)	92.55% (89.15–94.94%)	92.24% (88.79–94.69%)	93.48% (90.24–95.70%)	94.41% (91.34–96.44%)	91.18% (84.08–95.29%)	90.20% (82.89–94.59%)	92.16% (85.28–95.97%)	88.24% (80.55–93.14%)	91.18% (84.08–95.29%)	92.16% (85.28–95.97%)	93.14% (86.51–96.64%)	93.14% (86.51–96.64%)
PPV (95% CI)	96.08% (93.53–97.65%)	93.84% (90.77–95.94%)	92.51% (89.18–94.88%)	93.10% (89.94–95.32%)	92.57% (89.18––94.96%)	91.55% (87.83–94.21%)	93.56% (90.35–95.75%)	94.63% (91.67–96.57%)	92.62% (86.57–96.07%)	91.53% (85.10–95.33%)	92.79% (86.42–96.30%)	89.83% (83.06–94.09%)	91.82% (85.18–95.64%)	92.45% (85.81–96.13%)	93.81% (87.76–96.97%)	93.64% (87.44–96.88%)
NPV (95% CI)	98.72% (96.75–99.50%)	91.77% (88.29–94.28%)	88.66% (84.81–91.62%)	92.83% (89.48–95.18%)	86.13% (82.09–89.37%)	79.62% (75.24–83.40%)	87.76% (83.86–90.81%)	91.02% (87.47–93.64%)	92.08% (85.14–95.93%)	87.62% (79.96–92.62%)	83.93% (76.02–89.59%)	85.71% (77.76–91.15%)	82.30% (74.24–88.24%)	80.34% (72.23–86.53%)	86.36% (78.71–91.56%)	84.07% (76.22–89.68%)
PLR (95% CI)	22.73 (13.62–37.95)	14.14 (9.34–21.41)	11.47 (7.86–16.74)	12.53 (8.52–18.43)	11.56 (7.85–17.03)	10.06 (6.88–14.72)	13.48 (8.90–20.42)	16.34 (10.42–25.63)	10.58 (5.66–19.79)	9.10 (5.04–16.45)	10.85 (5.56–21.20)	7.45 (4.36–12.72)	9.46 (5.04–17.74)	10.33 (5.28–20.20)	12.77 (6.23–26.17)	12.40 (6.04–25.45)
NLR (95% CI)	0.01 (0.00–0.03)	0.08 (0.06–0.12)	0.12 (0.09–0.16)	0.07 (0.05–0.11)	0.15 (0.11–0.19)	0.24 (0.19–0.29)	0.13 (0.10–0.17)	0.09 (0.06–0.13)	0.07 (0.04–0.14)	0.12 (0.07–0.20)	0.16 (0.11–0.25)	0.14 (0.09–0.23)	0.18 (0.12–0.27)	0.21 (0.14–0.30)	0.13 (0.08–0.21)	0.16 (0.10–0.25)
Accuracy (95% CI)	97.31% (95.79–98.29%)	92.83% (90.62–94.55%)	90.58% (88.13–92.57%)	92.97% (90.78–94.68%)	89.24% (86.66–91.37%)	84.90% (81.99–87.42%)	90.58% (88.13–92.57%)	92.83% (90.62–94.55%)	92.38% (88.13–95.19%)	89.69% (85.00–93.03%)	88.34% (83.47–91.92%)	87.89% (82.96–91.54%)	87.00% (81.95–90.79%)	86.10% (80.94–90.03%)	90.13% (85.52–93.39%)	88.79% (83.97–92.29%)

ATB: active tuberculosis; LTBI: latent tuberculosis infection; AUC: area under the ROC curve; PPV: positive predictive value; NPV: negative predictive value; PLR: positive likelihood ratio; NLR: negative likelihood ratio; CI: confidence interval

### Validation of diagnostic models in another cohort

**Fig. 4:**
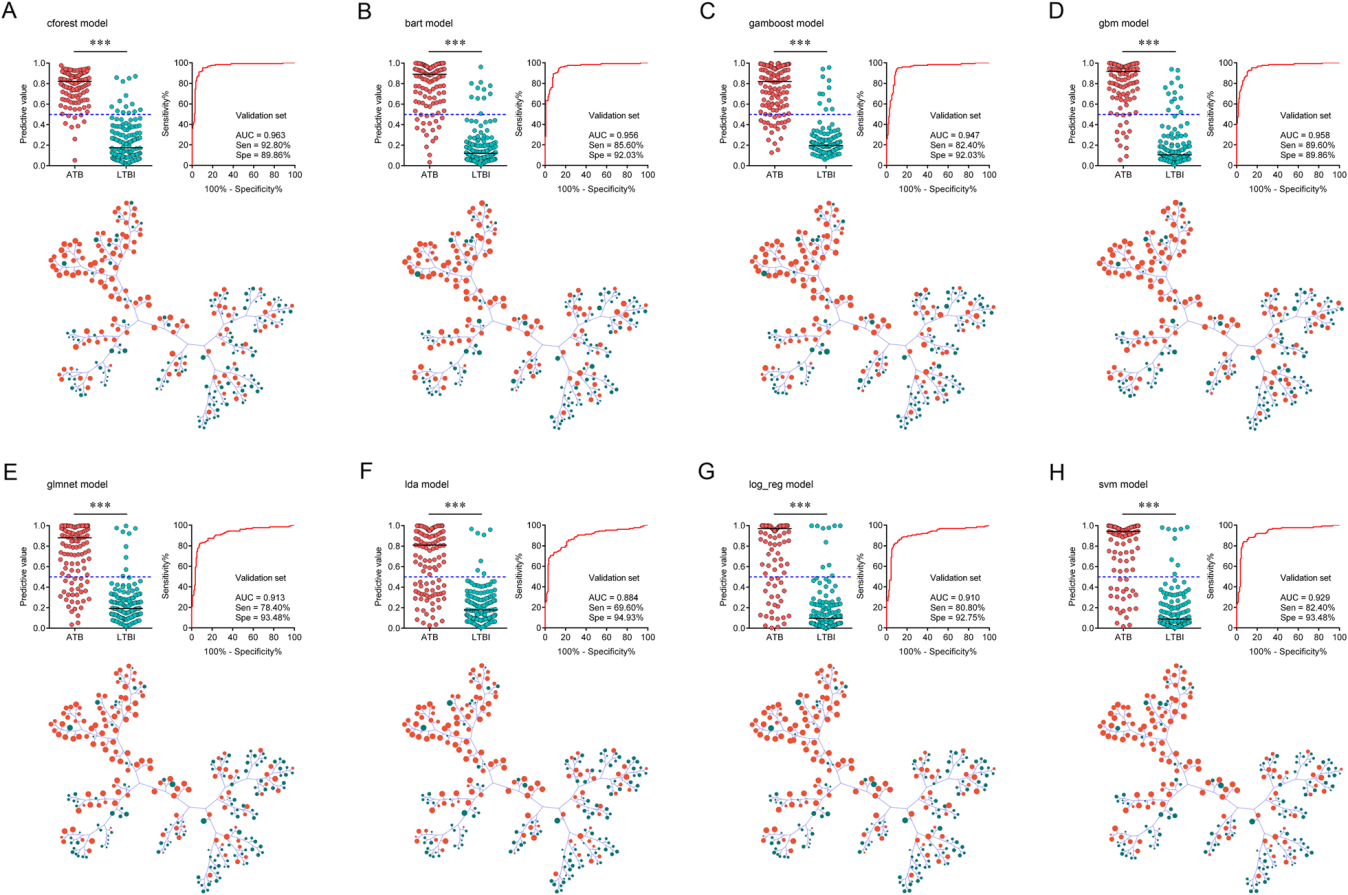
The validation of diagnostic models established for discriminating ATB patients from LTBI individuals. Scatter plots showing predictive values of diagnostic models (**A** cforest; **B** bart; **C** gamboost; **D** gbm; **E** glmnet; **F** lda; **G** log_reg; **H** svm) in ATB patients and LTBI individuals. Horizontal lines indicate the median. ***P < 0.001 (Mann–Whitney *U* test). Blue dotted lines indicate the cutoff value (0.5) in segregating these two groups. ROC curves showing the performance of diagnostic models (**A** cforest; **B** bart; **C** gamboost; **D** gbm; **E** glmnet; **F** lda; **G** log_reg; **H** svm) in segregating ATB patients from LTBI individuals. Tree and leaf plots showing predictive value of each participant when displaying as cluster distribution. The size of circle represents the predictive value. ATB: active tuberculosis; LTBI: latent tuberculosis infection; ROC: receiver operator characteristics; AUC: area under the ROC curve

**Fig. 5:**
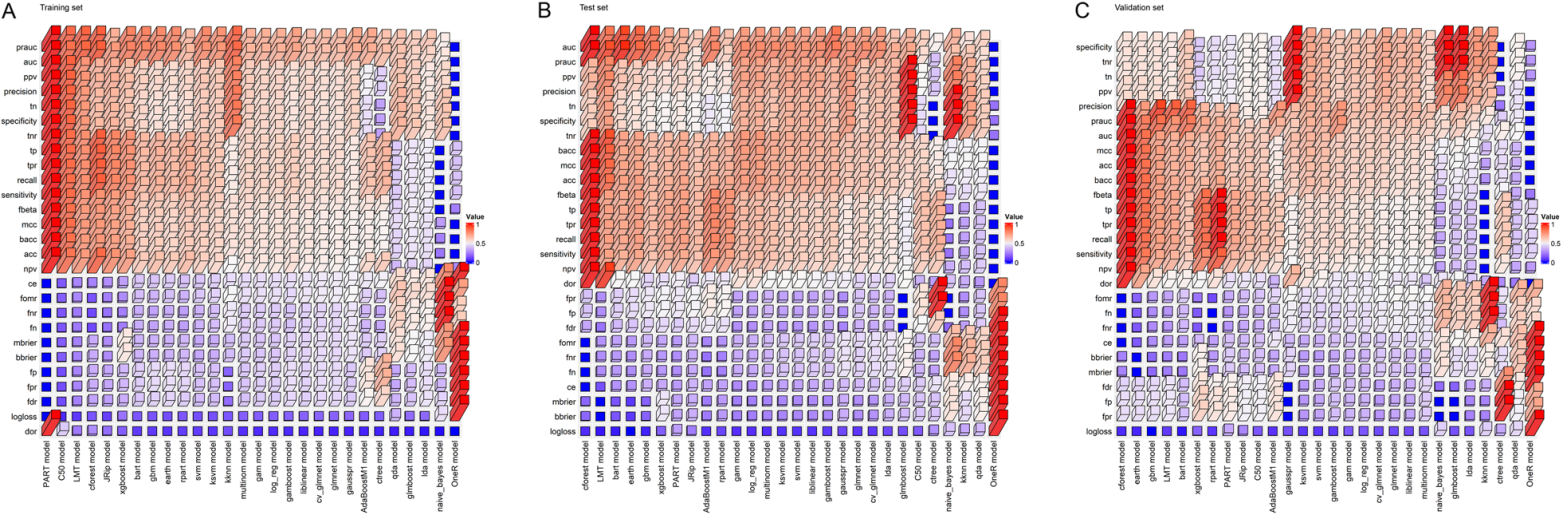
The diagnostic performance of the established 28 models for differentiating ATB patients from LTBI individuals in **A** training set, **B** test set, and **C** validation set. The height and color of the column represented the value of performance parameters after normalization to range between 0 and 1. acc: accuracy; auc: area under the ROC curve; bacc: balanced accuracy; bbrier: binary brier score; ce: classification error; dor: diagnostic odds ratio; fbeta: F-beta score; fdr: false discovery rate; fn: false negatives; fnr: false negative rate; fomr: false omission rate; fp: false positives; fpr: false positive rate, mbrier: multiclass brier score; mcc: matthews correlation coefficient; npv: negative predictive value; ppv: positive predictive value; prauc: area under the precision-recall curve; tn: true negatives; tnr: true negative rate; tp: true positives; tpr: true positive rate

An independent validation is indispensable for determining the robustness of a developed model based on machine learning. Therefore, another cohort (validation set) was included for the purpose in the study. Consistent with the observation in discovery cohort, cforest model exhibited significant discriminatory ability in validation cohort. Cforest model presented an AUC of 0.963 (95% CI 0.940–0.986) in validation set, with a sensitivity of 92.80% (95% CI 86.88–96.17%) and specificity of 89.86% (95% CI 83.69–93.86%) (Table 3, Fig. 4A). The utility of other models was summarized in Figs. 4, 5C, and Additional file 1: Fig. S1C.

**Table 3:** The performance of various models for segregating ATB from LTBI in validation cohort

Parameters	Validation set (n = 263, 125 ATB, 138 LTBI)							
	cforest	bart	gamboost	gbm	glmnet	lda	log_reg	svm
AUC (95% CI)	0.963 (0.940–0.986)	0.956 (0.932–0.981)	0.947 (0.919–0.975)	0.958 (0.935–0.981)	0.913 (0.876–0.950)	0.884 (0.841–0.927)	0.910 (0.872–0.949)	0.929 (0.896–0.962)
Sensitivity (95% CI)	92.80% (86.88–96.17%)	85.60% (78.38–90.69%)	82.40% (74.79–88.08%)	89.60% (83.02–93.82%)	78.40% (70.40–84.71%)	69.60% (61.05–76.98%)	80.80% (73.02–86.74%)	82.40% (74.79–88.08%)
Specificity (95% CI)	89.86% (83.69–93.86%)	92.03% (86.29–95.49%)	92.03% (86.29–95.49%)	89.86% (83.69–93.86%)	93.48% (88.07–96.53%)	94.93% (89.90–97.52%)	92.75% (87.18–96.02%)	93.48% (88.07–96.53%)
PPV (95% CI)	89.23% (82.73–93.48%)	90.68% (84.08–94.72%)	90.35% (83.55–94.53%)	88.89% (82.21–93.27%)	91.59% (84.78–95.51%)	92.55% (85.42–96.35%)	90.99% (84.21–95.03%)	91.96% (85.43–95.72%)
NPV (95% CI)	93.23% (87.64–96.40%)	87.59% (81.23–92.00%)	85.23% (78.66–90.04%)	90.51% (84.44–94.37%)	82.69% (75.99–87.82%)	77.51% (70.65–83.16%)	84.21% (77.58–89.15%)	85.43% (78.93–90.18%)
PLR (95% CI)	9.15 (5.55–15.07)	10.74 (6.06–19.02)	10.34 (5.83–18.33)	8.83 (5.36–14.56)	12.02 (6.35–22.76)	13.72 (6.61–28.50)	11.15 (6.10–20.38)	12.63 (6.68–23.89)
NLR (95% CI)	0.08 (0.04–0.15)	0.16 (0.10–0.24)	0.19 (0.13–0.28)	0.12 (0.07–0.19)	0.23 (0.16–0.32)	0.32 (0.24–0.42)	0.21 (0.14–0.30)	0.19 (0.13–0.28)
Accuracy (95% CI)	91.25% (87.22–94.10%)	88.97% (84.61–92.21%)	87.45% (82.90–90.92%)	89.73% (85.48–92.85%)	86.31% (81.63–89.95%)	82.89% (77.87–86.96%)	87.07% (82.48–90.60%)	88.21% (83.76–91.57%)

ATB: active tuberculosis; LTBI: latent tuberculosis infection; AUC: area under the ROC curve; PPV: positive predictive value; NPV: negative predictive value; PLR: positive likelihood ratio; NLR: negative likelihood ratio; CI: confidence interval

## Discussion

It is a growing notion that single biomarker is insufficient for differentiating Mtb infection status, while the powerful combination of multiple indicators would be trend for enhancing the utility [24, 25]. Nonetheless, the loss of diagnostic performance attributed to the unreasonable combination of data is usually easily neglected. There are many reasons for this outcome, including the researchers’ lack of perception over data characteristics as well as inappropriate selection of approaches for modelling. Although previous studies have explored the difference in many indexes for TB diagnostics, poor data utilization might exist in the combination of them. In recent years, with the in-depth study of multidimensional data analysis, algorithm-based machine learning shines brilliantly, especially in the field with the classification as the core [26, 27]. Therefore, it is a priority to rationally use algorithms to maximize diagnostic performance on multidimensional data. On the basis of the entry point, the present study investigated the potential of diagnostic models established using various algorithms involved in machine learning for segregating ATB from LTBI.

The study population contained two cohorts. One cohort was included as a discovery resource to develop diagnostic models using machine learning for differentiating ATB from LTBI, whereas another one was enrolled to validate the performance and availability the established models. The included indicators cover TB-specific immunological test (T-SPOT), non-specific immunological features (lymphocyte subset number and IFN-γ secretion ability), and routine laboratory tests. Therefore, our findings are relatively highly credible, inclusive and generalizable. Cforest model presented excellent performance in both discovery and validation cohort. The AUCs more than 0.96 in both test and validation set evidenced the potential diagnostic value of cforest model for differentiating ATB from LTBI. Cforest is a random forest algorithm based on conditional inference trees. It is a fast-learning rule that combines multiple decision trees together. Moreover, it can balance the errors of the data and generate classifiers with high accuracy. Remarkably, we found that cforest model outperformed log_reg model that was usually used in most previous studies (Z = 2.254, *P* = 0.024). This evidence suggested that the insufficient data value mining existed in many studies. Therefore, rational use of artificial intelligence in medical decision might be a developmental trend of precision medicine in the future. In addition, many of these models were comparable in terms of AUC. Meanwhile, there is a strong positive correlation in predictive values among various models (Additional file 2: Fig. S2). This observation indicated that the predictive trends were basically consistent across almost all models. However, there were subtle differences in data integration.

It was observed that CFP-10 SFC, ESAT-6 SFC, CD4 cell IFN-γ secretion, CD4 cell number, CD8 cell IFN-γ secretion, and FIB were dominant in contributing to the performance of many models including cforest model, bart model, gamboost model, gbm model and glmnet model. This finding denoted that complementary effect exists between specific and non-specific immune response in improving the diagnostic performance, while routine laboratory test could stabilize and locally optimize the model. Thus, most indicators of little significance when used separately could play a large or small role in constructing the model. Actually, this is also the advantage of machine learning. An appropriate and ideal algorithm could fully exploit the value of each data while avoiding overfitting.

Some points should be mentioned in this study. The development of algorithms used for classification is rapid. The current study comprehensively attempted the learners involved in “mlr3” as well as its auxiliary packages. The obtained results denoted that the models built based on these algorithms could be basically used for the effective diagnosis of TB. Nevertheless, cforest model performed better than the others in terms of performance. It means that various algorithms provide inconsistent advantages for classification under different data condition. On the one hand, the reasonable application of algorithm is based on the design of the algorithm itself. On the other hand, it also depends on the characteristics of the data, including the dimension of the data and the correlation between each other. The phenomenon suggests that more comprehensive consideration should be implemented in combining test data to maximize the efficiency for the diagnosis and prognosis of TB in the future.

On the whole, our model employed TB-specific and non-specific immunological indicators, as well as multi-dimensional routine laboratory tests (blood routine examination, biochemistry, coagulation, inflammatory reaction). These detections were usually available and could represent the host characteristics under Mtb infection in relatively comprehensive dimensions. Meanwhile, the reasonable use of machine learning algorithm and discovery-validation design involved in this study support the excellent performance and robustness of the model. Although the current trend is towards to POC test, the model established in the present study could still serve as an auxiliary or supplementary tool in TB diagnosis since it could be generated by the quick combination of the existing indicators. Therefore, the established model would be advantageous in clinical application.

Several limitations should be mentioned in the study. First, although the present study enrolled cohorts from two centers, the sample size in each center was limited. Thus, the robustness of the model built through machine learning needs further validation with large sample size to seek the applicability of the model. Second, since the existence of ATB patients with negative T-SPOT results has been reported by many studies [28–30], the lack of these cases in the current study might influence the performance of the established model. Therefore, more validation should be performed to access the efficacy of the model in the future. Third, given that fact that all participants in the current study were enrolled from a hospital setting, there would be some selection biases, in particular for LTBI individuals. Further inclusion in a community setting is needed to reduce selection biases and determine the efficiency of the established model more precisely. Fourth, the classification of Mtb infection status became more detailed in recent years, especially for the subclinical TB [31, 32]. Our study only classified the participants into ATB and LTBI. Thus, the more precise classification is required when developing diagnostic model in the future. Fifth, the advantage of machine learning usually exhibited its advantage under large amounts of dimensions. In spite of dozens of indicators included in our study, more emerging potential indicators, especially involved in omics [33–36] and flow cytometry [37], should be incorporated in the future to further strengthen the diagnostic performance of model. Finally, in addition to data itself, the parameter regulation can also affect the utility of the model, Therefore, more optimized algorithm and parameter setting should be further developed to achieve the maximum diagnostic efficacy in the future.

## Conclusions

Overall, the present study highlights the potential of cforest model based on laboratory data as a useful and anticipated tool in identifying Mtb infection status. Besides, it could serve as a tool to complement pathogenic detection to achieve ATB diagnosis in clinical setting. Furthermore, the successful implementation of our study provides novel insights on the integration of data from different dimensions, and lays foundation for realizing the effective combination of laboratory data and emerging artificial intelligence for TB diagnosis.

## Supplementary Information

**Additional file 1: Figure S1.** Radar plot showing the performance parameters of 28 models after normalization. (**A**) training set. (**B**) test set. (**C**) validation set.

**Additional file 2: Figure S2.** Triangular chart showing the correlation between predictive values of various diagnostic models in (A) training set, (B) test set, and (C) validation set.

## Data Availability

The data that support the findings of this study are available from the corresponding author upon reasonable request.

## Abbreviations

ATB: Active tuberculosis
LTBI: Latent tuberculosis infection
AUC: Area under the receiver operating characteristic curve
ESAT-6: Early secreted antigenic target 6
CFP-10: Culture filtrate protein 10
PHA: Phytohemagglutinin
TB: Tuberculosis
Mtb: *Mycobacterium tuberculosis*
PBMC: Peripheral blood mononuclear cells
SFC: Spot-forming cells
ROC: Receiver operating characteristic
SD: Standard deviation
IQR: Interquartile range
PPV: Positive predictive value
NPV: Negative predictive value
PLR: Positive likelihood ratio
NLR: Negative likelihood ratio
CI: Confidence intervals
WBC: White blood cell count
NEUT: Neutrophil count
LYMPH: Lymphocyte count
MONO: Monocyte count
EO: Eosinophil count
BASO: Basophil count
RBC: Red blood cell count
HGB: Hemoglobin
HCT: Hematocrit
RDW_CV: Coefficient variation of red blood cell volume distribution width
RDW_SD: Standard deviation in red cell distribution width
PLT: Platelet count
P_LCR: Platelet larger cell ratio
PCT: Plateletcrit
PDW: Platelet distribution width
TP: Total protein
ALB: Albumin
GLB: Globulin
T_CHOL: Total cholesterol
TG: Triglyceride
Ca: Calcium
Cl: Chlorine
K: Kalium
Na: Natrium
P: Phosphor
Mg: Magnesium
HsCRP: Hypersensitive C-reactive protein
APTT: Activated partial thromboplastic time
FIB: Fibrinogen
PT: Prothrombin time
TT: Thrombin time
D_D: D-Dimer
ESR: Erythrocyte sedimentation rate
PCA: Principal components analysis
t-SNE: T-distributed stochastic neighbor embedding
UMAP: Uniform manifold approximation and projection
