# Supplementary material for: Development of diagnostic algorithm using machine learning for distinguishing between active tuberculosis and latent tuberculosis infection
Source: BMC Infect Dis. 2022 Dec 29;22:965. doi: 10.1186/s12879-022-07954-7 (PMC9798640; doi:10.1186/s12879-022-07954-7)
Supplement: Supplementary file 1 — Additional file 1: Figure S1. Radar plot showing the performance parameters of 28 models after normalization. (A) training set. (B) test set. (C) validation set. [file 12879_2022_7954_MOESM1_ESM.pdf]

Supplementary Figure 1

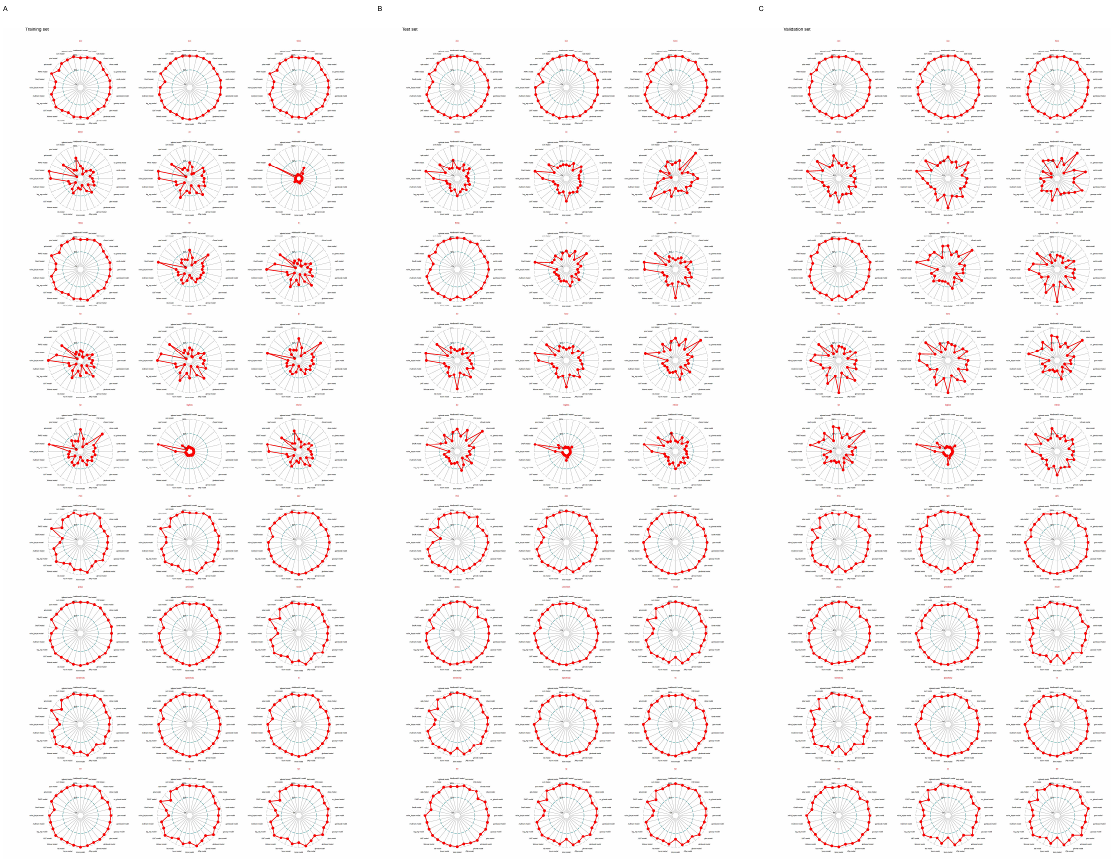

Supplementary Figure 1. Radar plot showing the performance parameters of 28 models after normalization. (**A**) training set. (**B**) test set. (**C**) validation set.
