# Supplementary material for: Development of diagnostic algorithm using machine learning for distinguishing between active tuberculosis and latent tuberculosis infection
Source: BMC Infect Dis. 2022 Dec 29;22:965. doi: 10.1186/s12879-022-07954-7 (PMC9798640; doi:10.1186/s12879-022-07954-7)
Supplement: Supplementary file 2 — Additional file 2: Figure S2. Triangular chart showing the correlation between predictive values of various diagnostic models in (A) training set, (B) test set, and (C) validation set. [file 12879_2022_7954_MOESM2_ESM.pdf]

Supplementary Figure 2

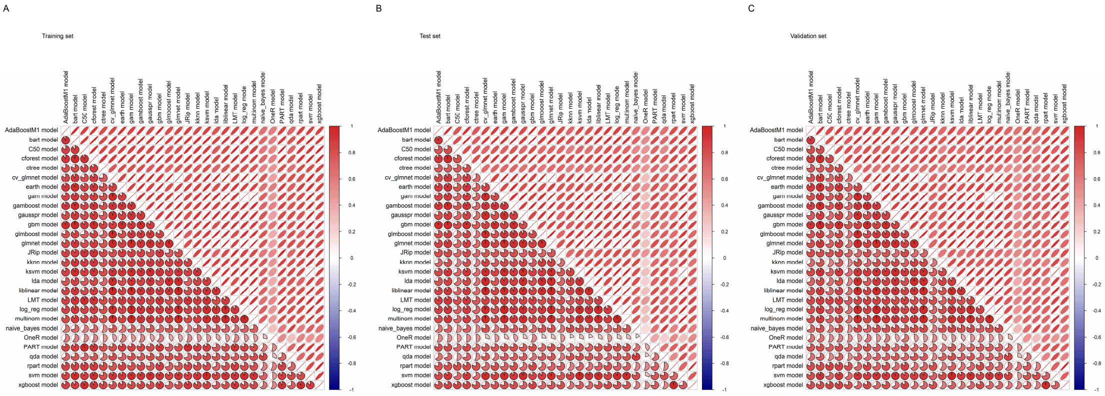

Supplementary Figure 2. Triangular chart showing the correlation between predictive values of various diagnostic models in (**A**) training set, (**B**) test set, and (**C**) validation set.
